# Multi-Sensor Vibration Signal Based Three-Stage Fault Prediction for Rotating Mechanical Equipment

**DOI:** 10.3390/e24020164

**Published:** 2022-01-21

**Authors:** Huaqing Peng, Heng Li, Yu Zhang, Siyuan Wang, Kai Gu, Mifeng Ren

**Affiliations:** 1 State Key Laboratory of Nuclear Power Safety Monitoring Technology and Equipment, China Nuclear Power Engineering Co., Ltd., Shenzhen 518172, China; penghuaqing@cgnpc.com.cn (H.P.); liheng@cgnpc.com.cn (H.L.); Yu.Zhang@cgnpc.com.cn (Y.Z.); 2 College of Electrical and Power Engineering, Taiyuan University of Technology, Taiyuan 030024, China; wangsiyuan0388@link.tyut.edu.cn

**Keywords:** vibration signal, fault prediction, multiple sensors, CNN, attention-Bi-LSTM

## Abstract

In order to reduce maintenance costs and avoid safety accidents, it is of great significance to carry out fault prediction to reasonably arrange maintenance plans for rotating mechanical equipment. At present, the relevant research mainly focuses on fault diagnosis and remaining useful life (RUL) predictions, which cannot provide information on the specific health condition and fault types of rotating mechanical equipment in advance. In this paper, a novel three-stage fault prediction method is presented to realize the identification of the degradation period and the type of failure simultaneously. Firstly, based on the vibration signals from multiple sensors, a convolutional neural network (CNN) and long short-term memory (LSTM) network are combined to extract the spatiotemporal features of the degradation period and fault type by means of the cross-entropy loss function. Then, to predict the degradation trend and the type of failure, the attention-bidirectional (Bi)-LSTM network is used as the regression model to predict the future trend of features. Furthermore, the predicted features are given to the support vector classification (SVC) model to identify the specific degradation period and fault type, which can eventually realize a comprehensive fault prediction. Finally, the NSF I/UCR Center for Intelligent Maintenance Systems (IMS) dataset is used to verify the feasibility and efficiency of the proposed fault prediction method.

## 1. Introduction

In the production processes of modern industries, the performance of rotating mechanical equipment may degrade over time, even resulting in failure due to long-term operation under severe conditions such as high speed, high temperature, high pressure, and heavy loads. To ensure the safety and efficiency of the operation, health monitoring and the establishment of a maintenance strategy have become active research focus in both industry and academia [1,2,3,4]. Initially, the maintenance strategy was implemented after fault diagnosis or preventive maintenance. We know that different types of equipment faults have specific vibration frequency characteristics. Therefore, traditional signal processing methods, such as Fourier transform (FT) [5], short-time Fourier transform (STFT) [6], and wavelet transform (WT) [7], have been proposed to obtain useful features from the vibration signal to reflect the operating status of the rotating mechanical equipment. However, the above fault diagnosis methods rely heavily on expert experience. In order to solve this problem, deep learning methods, such as CNN [8], LSTM [9], and the combined CNN and LSTM [10], have been used in fault diagnosis more recently, displaying the ability of deep feature self-learning without relying on manual intervention and prior knowledge. Although these methods can achieve excellent results in fault diagnosis, they cannot provide early warnings and take recovery measures before the fault occurs. Therefore, fault prediction is gradually emerging as a preventive maintenance method.

In [11], Peng Y et al. pointed out that fault prediction involves determining the RUL or working time of the diagnostic component based on the historical condition of the component. The research on RUL prediction can be divided into two categories: model-based methods and data-driven methods [12]. Lei Y et al. used maximum likelihood estimation and a particle filter algorithm to predict the RUL of bearings [13]. The proposed method is not well applicable to abrupt degeneration trends. Model-based methods rely on prior knowledge and specific conditions, whereas the data-driven approach attempts to use deep learning to deduce the degradation of equipment based on a large amount of historical data. In [14], a spectrum-principal-energy-vector method was used to extract features first, and then a deep CNN was formulated to obtain the RUL of bearings according to the features. For the first time, Xia Mei et al. divided the monitoring data into different health stages [15]. Based on this approach, the RUL of equipment was predicted using de-noising auto-encoder-based deep neural networks (DNNs). Although the above RUL methods can estimate how long it is until a fault will occur based on historical information, they are unable to provide the exact degradation period and fault type. To solve this problem, based on gray relational analysis, Wei X U et al. used a neural network model to predict the future state of a rolling bearing [16]. In [17], Xu H et al. used two models: a regression model and a classification model, which could not only predict the stage of degradation, but also classify the type of fault that would occur. However, the traditional wavelet packet transform (WPT) method was used to extract the time-frequency domain features of the original vibration signal, which requires expertise to select the appropriate basis function. Moreover, deep learning methods rely heavily on data information. Recent studies have shown that using multi-sensor data with sensor fusion technology can improve the accuracy and robustness of fault diagnosis models [18,19].

Therefore, in this paper, CNN, LSTM, and support vector classification (SVC) are combined to establish a novel three-stage fault prediction model for rotating mechanical equipment based on vibration signals from multiple sensors. Compared with the existing fault prediction results, the main contributions of the paper are as follows:

1. More formative vibration signals, used for training the fault prediction model, are collected from multiple sensors to improve the accuracy of the prediction method;
2. Deep features of varies degradation periods and fault types can be extracted by CNN and LSTM automatically without relying on manual intervention and professional knowledge;
3. The degradation period and fault type can be predicted simultaneously in advance with high accuracy.

The rest of this paper is structured as follows. In Section 2, the proposed three-stage fault prediction framework is generally introduced. Section 3 presents the details of the combined CNN and LSTM feature extraction, the attention-bidirectional (Bi)-LSTM regression model and the SVC classification mode. In Section 4, the superiority of the proposed fault prediction method is verified by applying it to the Intelligent Maintenance Systems (IMS) dataset. Section 5 concludes this paper.

## 2. Problem Formulation and Main Fault Prediction Framework

**Figure 1:**
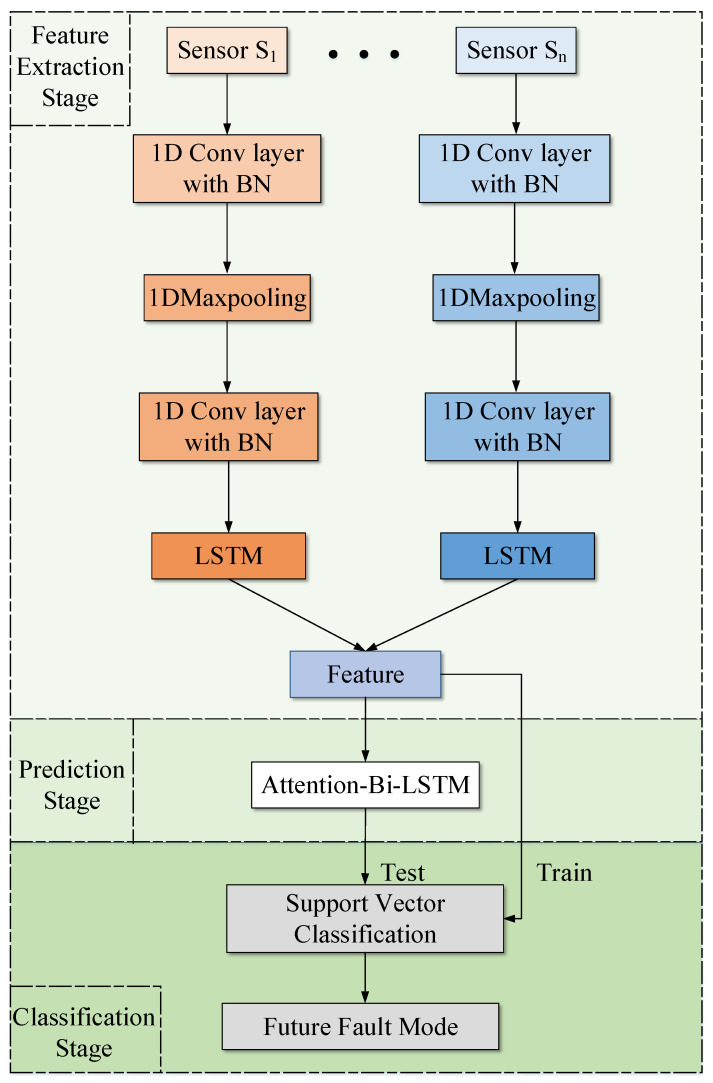
The overall framework of the three-stage failure prediction method.

To accomplish the tasks of predicting the degradation period and the type of failure, a novel three-stage fault prediction framework is presented based on the multi-sensor vibration signal, using deep learning and machine learning. The proposed architecture is illustrated in Figure 1, and can be divided into three parts: CNN-LSTM-based feature extraction, attention-Bi-LSTM-based prediction, and SVC-based classification.

As shown in Figure 1, the design of three-stage architecture is related to three objectives:

1. In the feature extraction stage, the original vibration signals collected by multiple sensors are sent to the CNN-LSTM network for the extraction of spatiotemporal features, which contain operating status information;
2. In the prediction stage, the attention-Bi-LSTM is trained to predict the trend of the features;
3. In the classification stage, based on the spatiotemporal features and their trends, the SVC model is formulated to identify the degradation period and the future fault type.

The original vibration signal from multiple sensors is collected first. In order to extract the spatiotemporal information of the obtained vibration signal, a CNN with a convolution-pooling-convolution structure and an LSTM network are combined to formulate the feature extraction model. Then, an attention-Bi-LSTM network is used to predict the feature trends, which can reflect the future health state of the rotating mechanical equipment. Finally, the predicted features are sent into the SVC for classification, which can achieve the purpose of predicting the degradation period and fault type simultaneously. The emtire process of the proposed three-stage fault prediction method is detailed in the following section.

## 3. Deep Learning Network-Based Three-Stage Fault Prediction

### 3.1. Feature Extraction Stage

**Figure 2:**
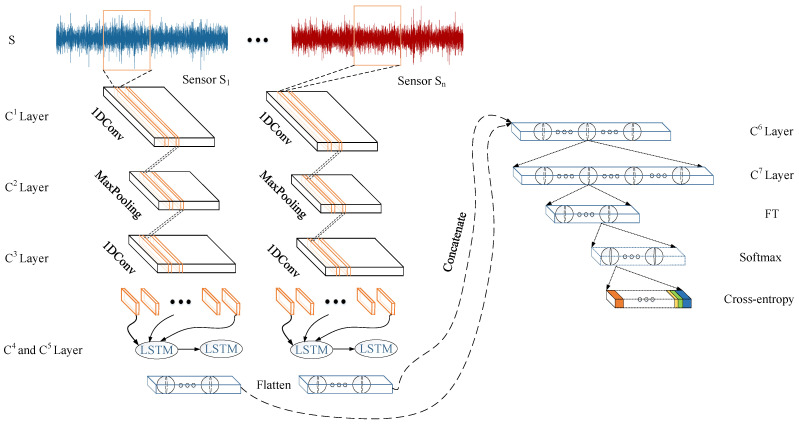
The model structure of the feature extraction model ($C^{i}$ denotes the output of the *i*th layer; FT denotes the output of the last dense layer).

Vibration signals are collected by multiple sensors is in the form of time series with noise. This is susceptible to the amplitude. Therefore, it is necessary to perform feature extraction on the time-domain vibration signals. The CNN can extract the features of the original vibration signal, reducing the amount of data and diminishing the noise. However, it only can capture the spatial features, and the temporal features are ignored. Therefore, the CNN-LSTM is used in this paper to extract the spatiotemporal features of the original vibration signal. The framework of the CNN-LSTM model is illustrated in Figure 2. First, a CNN with a convolution-pooling-convolution structure is used to extract the spatial features of the original vibration signal. Then, the deep abstract of temporal features can be obtained by adding the LSTM network after the CNN.

We can denote the originally collected vibration signal in the form of a time series from the *m*th sensor as $V^{m}={\left\{V_{t}^{m}\right\}}_{m=1,2,\cdots ,n;t=1,2,\cdots }$. In order to deal with the problem of sample imbalance and obtain more detailed characteristics of the vibration signal, the data are divided into windows. At the same time, it is necessary to ensure that the window size is properly chosen. For the data in each window, 1D convolution is first used to extract the shallow features. The convolution operation formula of the τth window is as follows:

(1) $$C_{i}^{1}\left(\tau \right)=f\left(\sum_{i=1}^{L}\omega_{i}^{1}*V^{m}\left(\tau \right)+b_{i}^{1}\right),\left(i=1,2,\cdots ,L\right)$$

where $C_{i}^{1}\left(\tau \right)$ is named the feature map, and it denotes the *i*th channel output of the convolution operation; *L* is the total number of channels; $V^{m}\left(\tau \right)$ denotes the vibration signals in the τth window; * denotes the dot product; f(·) is the activation function; *N* represents the number of convolution kernels; $\omega_{i}^{1}$ is the *i*th weight parameters of the 1st layer; $b_{i}^{1}$ represents the *i*th bias of the 1st layer; and ω and *b* are undetermined parameters to be trained.

After the 1D convolution operations, 1D maxpooling operations for the processing results ${\left\{C_{i}^{1}\right\}}_{i=1,2,\ldots ,L}$ are performed, which can reduce the dimension of the feature maps and further extract key features of the vibration signal:

(2) $$C_{i}^{2}=\max\left\{C_{i,1}^{1},C_{i,2}^{1},\cdots ,C_{i,d}^{1}\right\},\left(i=1,2,\cdots ,L\right)$$

where $C_{i,k}^{1}$ represents the *k*th value of the *i*th channel of the 1st layer, *d* is the number of samples participating in the maxpooling operation, and $C_{i}^{2}$ denotes the output of the pooling layer.

Another convolution operation is performed on the processing result of the pooling operation in order to extract the deeper features of the vibration signal:

(3) $$C_{l}^{3}\left(\tau \right)=f\left(\sum_{l=1}^{N}\omega_{l}^{3}*C^{2,l}\left(\tau \right)+b_{l}^{3}\right)$$

where the symbols in (3) are the same as those in (1).

Considering the time characteristics of the vibration signal, the LSTM neural network is used to extract the temporal features, processing the series data with the forgetting memory scheme while avoiding the gradient disappearance and gradient explosion problems. LSTM realizes the function of long-term and short-term state transfer through the structure of an input gate, forget gate, and output gate. After obtaining the spatial features from the CNN ${\left\{C_{l}^{3}\right\}}_{l=1,2,\ldots }$, two LSTM networks are employed to extract the temporal features.The operation principle is as follows: the update of the hidden state $h^{l}$ at time *l* is based on the joint action of the input gate $i^{l}$, forgetting gate $f^{l}$, output gate $o^{l}$, cell state $c^{l}$, and hidden state $h^{l-1}$ at time l−1. The specific formula is as follows:

(4) $$\begin{array}{cc} \; & i^{l}=\sigma \left(W^{1i}C_{l}^{3}+W^{2i}h^{l-1}+b^{i}\right),\\ \; & f^{l}=\sigma \left(W^{1f}C_{l}^{3}+W^{2f}h^{l-1}+b^{f}\right),\\ \; & o^{l}=\sigma \left(W^{1o}C_{l}^{3}+W^{2o}h^{l-1}+b^{o}\right),\\ \; & c^{l}=f^{l}⊙c^{l-1}+i^{l}⊙\tanh\left(W^{1c}C_{l}^{3}+W^{2c}h^{l-1}+b^{c}\right),\\ \; & h^{l}=o^{l}⊙\tanh\left(c^{l}\right). \end{array}$$

where *W* denotes weight and *b* is bias. σ denotes the sigmoid activation function, and ⊙ is Hadamard product.

We can input the LSTM-processed features ${\left\{h^{l}\right\}}_{l=1,2,\ldots }$ into the two fully connected layers to obtain the eventual features of the original vibration signal, which can be denoted as ${\left\{FT^{l}\right\}}_{l=1,2,\ldots }$.

(5) $$FE^{l}=f\left(\omega_{l}\cdot h^{l}+b_{l}\right)$$

(6) $$FT^{l}=f\left(\omega_{l}^{\prime }\cdot FE^{l}+b_{l}^{\prime }\right)$$

where ω and $\omega^{\prime }$ denote weights, *b* and $b^{\prime }$ are biases, and f(·) is the activation function.

We can iput the obtained feature ${\left\{FT^{l}\right\}}_{l=1,2,\ldots }$ into the Softmax layer to obtain the probability that the current feature belongs to various categories $\hat{p}$. Then, one can input the probability $\hat{p}$ into the cross-entropy loss function to complete the entire back-propagation process. The cross-entropy loss function is defined as follows:

(7) $$L=-\sum_{i=1}^{N}\left[p^{\left(i\right)}\log{\hat{p}}^{\left(i\right)}+\left(1-p^{\left(i\right)}\right)\log\left(1-{\hat{p}}^{\left(i\right)}\right)\right]$$

where $p^{\left(i\right)}$ denotes the true probability that $V^{m}={\left\{V_{t}^{m}\right\}}_{m=1,2,\cdots ,n;t=1,2,\cdots }$ belongs to the *i*th category; and ${\hat{p}}^{\left(i\right)}$ denotes the corresponding probability obtained from the deep networks’ classification result. The Softmax layer and the cross-entropy loss function are used here to provide a target for the back-propagation process of the deep network. It can endow classification attributes to the extracted features.

### 3.2. Prediction Stage

**Figure 3:**
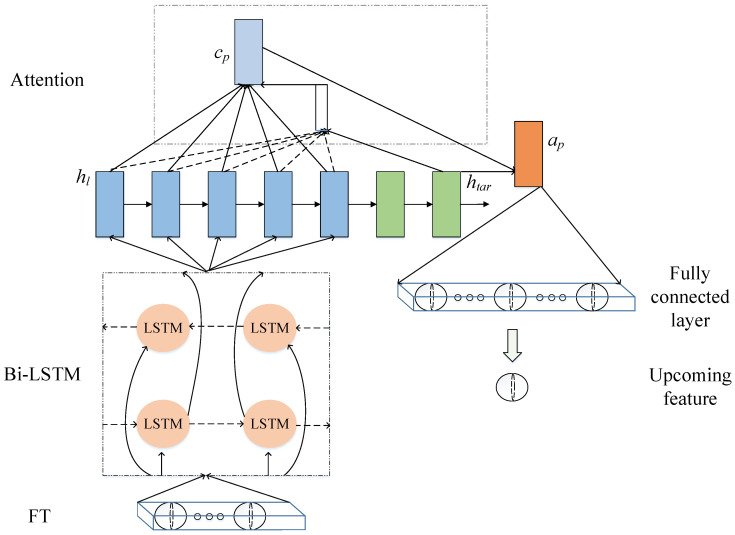
Flow chart of the feature prediction stage.

In order to achieve the purpose of failure prediction, after obtaining the operating state characteristics of the rotating machinery, the feature trend should be predicted. Since these features have a time series correlation, attention Bi-LSTM is used as the prediction model here, as shown in Figure 3. Bi-LSTM consists of a forward LSTM and a backward LSTM, which are able to capture features of both past and upcoming time series. In addition, in order to enhance the correlation between the output results of Bi-LSTM, an attention mechanism is added, which can achieve the purpose of redistributing the weights between the output results.

The features obtained from the feature extraction stage, ${\left\{FT^{l}\right\}}_{l=1,2,\ldots }$ are first sent into the Bi-LSTM network. The specific principle formulas are as follows:

(8) $$\begin{array}{cc} i_{F}^{l} & =\sigma \left(W_{F}^{1i}FT_{F}^{l}+W_{F}^{2i}h_{F}^{l-1}+b_{F}^{i}\right)\\ f_{F}^{l} & =\sigma \left(W_{F}^{1f}FT_{F}^{l}+W_{F}^{2f}h_{F}^{l-1}+b_{F}^{f}\right)\\ o_{F}^{l} & =\sigma \left(W_{F}^{1o}FT_{F}^{l}+W_{F}^{2f}h_{F}^{l-1}+b_{F}^{o}\right)\\ c_{F}^{l} & =f_{F}^{l}⊙c_{F}^{l-1}+i_{F}^{l}⊙\tanh\left(W_{F}^{1c}FT_{F}^{l}+W_{F}^{2c}h_{F}^{l-1}+b_{F}^{c}\right)\\ h_{F}^{l} & =o_{F}^{l}⊙\tanh\left(c_{F}^{l}\right) \end{array}$$

(9) $$\begin{array}{cc} i_{B}^{l} & =\sigma \left(W_{B}^{1i}FT_{B}^{l}+W_{B}^{2i}h_{B}^{l+1}+b_{B}^{i}\right)\\ f_{B}^{l} & =\sigma \left(W_{B}^{1f}FT_{B}^{l}+W_{B}^{2f}h_{B}^{l+1}+b_{B}^{f}\right)\\ o_{B}^{l} & =\sigma \left(W_{B}^{1o}FT_{B}^{l}+W_{B}^{2f}h_{B}^{l+1}+b_{B}^{o}\right)\\ c_{B}^{l} & =f_{B}^{l}⊙c_{B}^{l+1}+i_{B}^{l}⊙\tanh\left(W_{B}^{1c}FT_{B}^{l}+W_{B}^{2c}h_{B}^{l+1}+b_{B}^{c}\right)\\ h_{B}^{l} & =o_{B}^{l}⊙\tanh\left(c_{B}^{l}\right) \end{array}$$

(10) $$h_{l}=\left[\begin{array}{cc} h_{F}^{l} & h_{B}^{l} \end{array}\right]$$

where the subscripts *F* and *B* denote forward and backward, respectively, and $h_{l}$ represents the concatenation of the forward output $h_{F}^{l}$ and backward output $h_{B}^{l}$ of Bi-LSTM.

The basic idea of adding the attention mechanism in Bi-LSTM is to calculate the correlation between the target hidden state $h_{tar}$ and the output hidden states $h_{l}$ of Bi-LSTM, and then to output the attention vector [20]. The principle formulas are listed as follows:

(11) $$\;\mathrm{score}\;\left(h_{tar},h_{l}\right)=h_{tar}^{T}Wh_{l}$$

(12) $$\alpha_{ts}=\frac{\exp\left(\mathrm{score}\left(h_{tar},h_{l}\right)\right)}{\sum_{l^{\prime }=1}^{L}\exp\left(\mathrm{score}\left(h_{tar},h_{l^{\prime }}\right)\right)}$$

(13) $$c_{p}=\sum_{s=1}^{S}\alpha_{ts}h_{l}$$

(14) $$a_{p}=\tanh\left(W_{c}\left[c_{p};h_{tar}\right]\right)$$

where score(·) is a function, $\alpha_{ts}$ is the attention weight, $c_{p}$ denotes the context vector, $a_{p}$ represents the attention vector, and *W* and $W_{c}$ denote weights.

We input the ${\left\{a_{p}\right\}}_{p=1,2,\ldots }$ into the two fully connected layers to obtain the result of the feature prediction stage, which we denote as ${\left\{ft^{p}\right\}}_{p=1,2,\ldots }$.

(15) $$fe^{p}=f\left(\omega_{p}\cdot a_{p}+b_{p}\right)$$

(16) $$ft^{p}=f\left(\omega_{p}^{\prime }\cdot a_{p}+b_{p}^{\prime }\right)$$

where ω and $\omega^{\prime }$ denote weights, *b* and $b^{\prime }$ are biases, and f(·) is the activation function.

### 3.3. Classification Stage

After obtaining the future spatiotemporal features of the original vibration signal using the attention-Bi-LSTM prediction model, the degradation period and fault type should be identified by formulating a classification model. In fact, the feature types are divided into several categories by training a Softmax classifier in the feature extraction stage. However, since the prediction step is added after the feature extraction step, the classification model and the prediction model cannot perform the same backpropagation. Therefore, considering the errors of the prediction results, the more robust SVC is used as the classifier instead of the Softmax with rigorous function mapping [21]. The basic idea of the SVC model is to find a classifier that maximizes the classification interval between the hyperplane and the support vector. The principle of the SVC can be addressed as follows. For the predicted features ${\left\{ft^{p}\right\}}_{p=1,2,\ldots }$, we denote their failure modes as ${\left\{y^{p}\right\}}_{p=1,2,\ldots },y\in \left\{-1,1\right\}$. The SVC problem can be converted into the following quadratic optimization problem:

(17) $$\mathrm{Max}\sum_{i=1}^{n}\alpha_{i}-\frac{1}{2}\sum_{i=1}^{n}\sum_{i=1}^{n}\alpha_{i}\alpha_{j}y_{i}y_{j}K\left(ft_{i}^{p},ft_{j}^{p}\right)$$

(18) $$\begin{array}{cc} \; & \;s.t.\;\alpha_{i}\geq 0,i=1,\cdots ,n\\ \; & \sum_{i=1}^{n}\alpha_{i}y_{i}=0 \end{array}$$

where α is the Lagrange multiplier. $K\left(ft_{i}^{p},ft_{j}^{p}\right)$ is the kernel function, which can map the linearly inseparable samples in the initial space to the linearly separable samples in the high-dimensional space. In this paper, we use the radial basis function (RBF) as the kernel function.

(19) $$K\left(ft_{i}^{p},ft_{j}^{p}\right)=e^{-\gamma ∥f_{i}^{p}-f_{j}^{p}{∥}^{2}},\gamma >0$$

where γ is the width of the RBF. The output function of the category can be obtained using the following formula:

(20) $$f\left(x\right)=\mathrm{sgn}\left[\sum_{i=1}^{n}\alpha_{i}^{*}y_{i}K\left(ft_{i}^{p},ft_{j}^{p}\right)+b^{*}\right]$$

where $b^{*}$ is the classification threshold, which is obtained by substituting support vectors.

The SVC fault diagnosis model in this paper adopts the One vs. Rest (OvR) approach to realize the multi-classification of faults. The main ideas of OvR are as follows: if *N* categories need to be classified, *N* binary classifiers should be constructed. In the training process, select one category as positive and the others as negative, and then classify them in turn. In the test process, the test samples are sequentially brought into the trained *N* classifiers for calculation, and then the final classification result can be calculated.

### 3.4. Implementing the Proposed Fault Prediction Strategy

At last, it is worth summarizing the pseudo-code of the entire fault prediction algorithm, which is shown in Algorithm 1:
**Algorithm 1** Fault prediction algorithm1:**procedure**training process2:  **Input:** original signal from sensor $S_{1}$, *…*,sensor $S_{n}$, and initial labels ${\left\{y_{m}\right\}}_{m=1,2,\ldots }$ which are fault modes3:  **Output:** the model parameters of trained Attention-Bi-LSTM and SVC; test dataset ${\left\{FT^{d}\right\}}_{d=1,2,\ldots }$4:  Random initialization: The feature extraction network parameters ${\left\{\omega \right\}}^{E}$ and ${\left\{b\right\}}^{E}$; The feature prediction network parameters ${\left\{W\right\}}^{P}$ and ${\left\{bi\right\}}^{P}$5:  Split original signal from $S_{1}$, *…*,sensor $S_{n}$ into {training set} and {test set} for feature extraction model6:  **for** sensor $S_{1},\ldots$, sensor $\left.S_{n}\right\}$ in {{training set},{test set}} **do**7:    **for** *k* in range(times),$times=\left\{\begin{array}{cc} \;\mathrm{epochs}\; & ,\mathrm{if}\left\{\mathrm{sensor}\;S_{1},\ldots ,\mathrm{sensor}\;S_{n}\right\}==\left\{\mathrm{training}\;\mathrm{set}\right\}\\ 1 & ,\mathrm{if}\left\{\mathrm{sensor}\;S_{1},\ldots ,\mathrm{sensor}\;S_{n}\right\}==\left\{\mathrm{test}\;\mathrm{set}\right\} \end{array}\right.$**do**8:            Calculate $1_{1}^{st}$ one-dimensional convolution layer for sensor $S_{1}$ as $C_{k}^{1}\left(S_{1}\right)$ based on $\omega_{1}^{1}$ and $b_{1}^{1}$;…;Calculate $1_{n}^{st}$ one-dimensional convolution layer for sensor $S_{n}$ as $C_{k}^{1}\left(S_{n}\right)$ based on $\omega_{n}^{1}$ and $b_{n}^{1}$9:            Calculate $2_{1}^{nd}$ one-dimensional maxpooling layer for $C_{k}^{1}\left(S_{1}\right)$ as $C_{k}^{2}\left(S_{1}\right)$;…;Calculate $2_{n}^{nd}$ one-dimensional maxpooling layer for $C_{k}^{1}\left(S_{n}\right)$ as $C_{k}^{2}\left(S_{n}\right)$10:          Calculate $3_{1}^{rd}$ one-dimensional convolution layer for $C_{k}^{2}\left(S_{1}\right)$ as $C_{k}^{3}\left(S_{1}\right)$ based on $\omega_{1}^{3}$ and $b_{1}^{3}$;…;Calculate $3_{n}^{rd}$ one-dimensional convolution layer for $C_{k}^{2}\left(S_{n}\right)$ as $C_{k}^{3}\left(S_{n}\right)$ based on $\omega_{n}^{3}$ and $b_{n}^{3}$11:          Calculate $4_{1}^{th}$ and $5_{1}^{th}$ LSTM layers for $C_{k}^{3}\left(S_{1}\right)$ as $C_{k}^{5}\left(S_{1}\right)$ based on $\omega_{1}^{4,5}$ and $b_{1}^{4,5}$; *…*;Calculate $4_{n}^{th}$ and $5_{n}^{th}$ LSTM layers for $C_{k}^{3}\left(S_{n}\right)$ as $C_{k}^{5}\left(S_{n}\right)$ based on $\omega_{n}^{4,5}$ and $b_{n}^{4,5}$12:          Concatenate $C_{k}^{5}\left(S_{1}\right)$, *…*, $C_{k}^{5}\left(S_{n}\right)$ as $C_{k}^{6}$13:          Calculate $1^{st}$ Dense layer for $C_{k}^{6}$ as $C_{k}^{7}$ and $2^{nd}$ Dense layer for $C_{k}^{7}$ as${\left\{FT^{m}\right\}}_{m=1,2,\ldots }=\left\{\begin{array}{cc} {\left\{FT^{tr}\right\}}_{tr=1,2,\ldots }, & \;\mathrm{if}\;\left\{\;\mathrm{sensor}\;S_{1},\ldots ,\;\mathrm{sensor}\;S_{n}\right\}==\left\{\;\mathrm{training}\;\mathrm{set}\;\right\}\\ {\left\{FT^{te}\right\}}_{te=1,2,\ldots }, & \;\mathrm{if}\;\left\{\;\mathrm{sensor}\;S_{1},\ldots ,\;\mathrm{sensor}\;S_{n}\right\}==\left\{\;\mathrm{test}\;\mathrm{set}\;\right\} \end{array}\right.$ based on $\omega^{7}$ and $b^{7}$, then input ${\left\{FT^{m}\right\}}_{m=1,2,\ldots }$ to softmax layer14:          Calculate the cross-entropy loss; Update the ${\left\{\omega \right\}}^{E}$ and ${\left\{b\right\}}^{E}$, i.e.,${\left\{\omega \right\}}^{E},{\left\{b\right\}}^{E}\leftarrow {\left\{\omega^{\prime }\right\}}^{E},{\left\{b^{\prime }\right\}}^{E}$15:    **end for**16:  **end for**17:  Reorder ${\left\{FT^{m}\right\}}_{m=1,2,\ldots }$ in time series18:  Divide ${\left\{FT^{m}\right\}}_{m=1,2,\ldots }$ to training dataset ${\left\{FT^{\mathrm{train},a}\right\}}_{a=1,2,\ldots }$ and test dataset ${\left\{FT^{\mathrm{test},d}\right\}}_{d=1,2,\ldots }$19:  **for** epoch in range (EPOCHS) **do**20:          Calculate the Bi-LSTM model for ${\left\{FT^{\mathrm{train},a}\right\}}_{a=1,2,\ldots }$ as $P_{k}^{1}$ based on $W^{1}$ and $bi^{1}$21:          Calculate the attention layer for $P_{k}^{1}$ as $P_{k}^{2}$ based on $W^{2}$ and $bi^{2}$22:          Calculate the Dense layer for $P_{k}^{2}$ as $P_{k}^{3}$ based on $W^{3}$ and $bi^{3}$23:          Calculate the RMSE loss function and update the ${\left\{W\right\}}^{P}$ and ${\left\{bi\right\}}^{P}$, i.e., ${\left\{W\right\}}^{P},{\left\{bi\right\}}^{P}\leftarrow {\left\{W^{\prime }\right\}}^{P},{\left\{bi^{\prime }\right\}}^{P}$24:  **end for**25:  Use cross-validation to train the SVC model with ${\left\{FT^{m}\right\}}_{m=1,2,\ldots }$ and ${\left\{{\hat{y}}_{m}\right\}}_{m=1,2,\ldots }$ to obtain the parameters of the SVC ${\left\{support\cdot vector\right\}}^{C}$26:**end procedure**27:**procedure**test process28:  **Input:** ${\left\{FT^{\mathrm{test},d}\right\}}_{d=1,2,\ldots }$ and ${\left\{y_{d}\right\}}_{d=1,2,\ldots }$ which are fault mode labels of ${\left\{FT^{\mathrm{test},d}\right\}}_{d=1,2,\ldots }$29:  **Output:** the labels ${\left\{{\hat{y}}_{d}\right\}}_{d=1,2,\ldots }$ of fault prediction and their accuracy30:  Leading-in the parameters of trained attention-Bi-LSTM model and SVC model31:  Calculate attention-Bi-LSTM model prediction results for ${\left\{FT^{\mathrm{test},d}\right\}}_{d=1,2,\ldots }$ as ${\left\{ft^{\mathrm{test},d}\right\}}_{d=1,2,\ldots }$32:  Calculate SVC classifying results ${\left\{{\hat{y}}_{d}\right\}}_{d=1,2,\ldots }$ for ${\left\{ft^{\mathrm{test},d}\right\}}_{d=1,2,\ldots }$33:  Calculate accuracy $=\frac{†\left[{\hat{y}}_{d}==y_{d}\right]}{†\left[y_{d}\right]},d=1,2,\ldots$, where †[x] denotes the number of *x*34:**end procedure**

## 4. Validating the Proposed Method

In order to verify the fault prediction method proposed in this paper, the IMS dataset was used [22]. The IMS dataset contains three data sets: dataset 1, with two acceleration sensors on each bearing, and dataset 2 and dataset 3, with one accelerometer on each bearing, respectively. In view of the fact that this experiment needs to predict the failure modes through multiple sensors, dataset 1 is used to verify the proposed method. The sampling frequency of dataset 1 is 20 kHz, and the sampling duration is 1 s. The sampling interval of the first 43 rounds of each acceleration sensor was used to collect data every 5 min, then to collect data every 10 min and generate a data file containing 20,480 sampling points. The Python programming environment was used, based on the Keras framework of version 2.3.1. All experiments were performed on Intel Xeon ES-2620 CPU.

### 4.1. The Description of the Dataset

At the end of the test-to-failure experiment, in dataset 1, bearing 3 displayed an inner race defect and bearing 4 displayed a roller element defect [23]. The purpose of this experiment was to classify the bearing degradation period and identify the early fault type of the bearing based on the vibration signals from multiple sensors. Therefore, bearings 1-3 and 1-4 were selected for research, as shown in Table 1. The entire life of the bearing was divided into four stages: the norm period, slight period, severe period, and failure period. In addition, there were two types of faults: the inner race defect and the roller element defect. Therefore, there were seven fault modes in the experiment, which were the norm period, the slight inner race defect, the severe inner race defect, the inner race failure, the slight roller element defect, the severe roller element defect, and the roller element failure.

**Figure 4:**
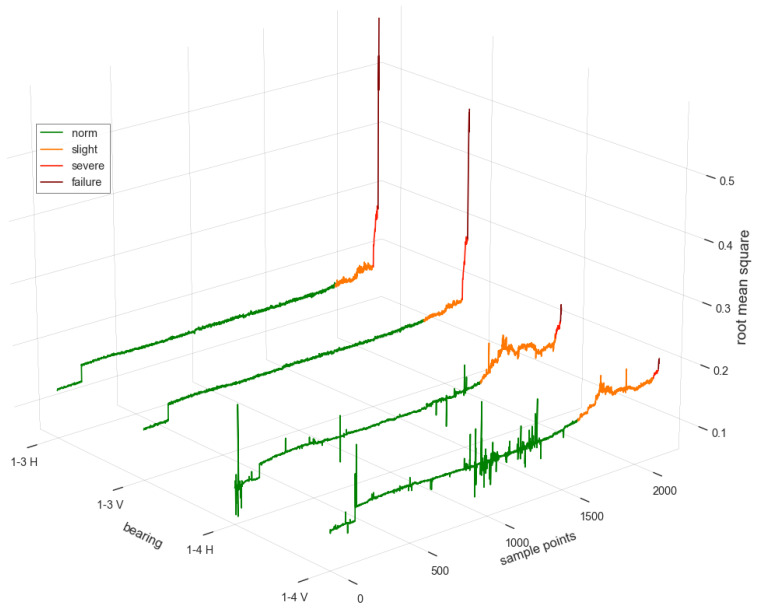
Root mean square features of each bearing.

The IMS dataset does not have a detailed true label of the degradation period and specific failure of the bearing. Therefore, according to the labeling method in [17,24], the threshold of each stage should be set according to actual needs.In this simulation, the root mean square (RMS) features of the 20,480 vibration signals collected per second from each sensor were first extracted. Then, expertise was involved in labeling the degradation period, which is shown in Figure 4 and Table 2. In our simulation, 20,480 samples of vibration signals were collected every 10 min, and these samples were saved in one file. There were 2156 sampling files in the whole life cycle. For example, the samples from the 2120th file to the 2151th file belonged to the severe period for bearing 1-3 H.

### 4.2. Feature Extraction

#### 4.2.1. Training Process

The data need to be processed appropriately in order to extract the more detailed characteristics of the vibration signal and train the better performance of fault prediction model. Using the trial and error method, the samples in each file with 20,480 samples were divided into 80 windows.Therefore, there were 256 samples in every window.

In the feature extraction stage, the task of the CNN-LSTM network is to effectively extract the spatiotemporal features of the degradation period and fault type simultaneously. The framework of the CNN-LSTM network for feature extraction in Figure 2 was used here. The network settings are shown in Table 3. The inputs of Sensor *H* and Sensor *V* were both N×256×1, with *N* being the number of windows. The batch size was 512. The number of epochs was 25, and the optimizer used was Adam. The cross-entropy in (7) is employed here as the loss function.

#### 4.2.2. Verifying the Validity of the Feature Extraction Model

In order to verify the effectiveness of the proposed multi-sensor CNN-LSTM feature extraction model, a CNN-LSTM network with a single sensor was used as the comparative model. The evaluation equation was chosen as follows:

(21) $$\mathrm{accuracy}=\frac{TP}{TP+FP}$$

where TP is the number of true positives and FP is the number of false positives. The comparison results between our model and other models are shown in Table 4:

From Table 4, it can be seen that the classification accuracy based on a single-sensor CNN-LSTM feature extraction model was lower than that based on the multi-sensor model. The reason is that the large fluctuation and noise of the vibration signals from a single sensor may mislead the identification of the degradation period, such as the fluctuation of 1-4V shown in Figure 4. However, the vibration signals from multiple sensors can provide more comprehensive information, which could improve the classification accuracy.

### 4.3. Trend Prediction

#### 4.3.1. Training Process

The input of the prediction model is the features obtained from the previous section. The characteristics of the normal period were not used to predict degradation trend, because the data in that period often show no trend information. In this simulation, the severe and failure periods for the inner race and the roller element were predicted, respectively. In order to illustrate the effectiveness of the proposed Attention Bi-LSTM model, only the prediction results of the failure period for roller defects are presented in the following as a representation of the model’s performance.The input of our training process was set at 1994×windowwidth×10, and the input of the testing process was 400×windowwidth×10. The parameter settings of the prediction model are shown in Table 5.

Sliding time window technology was used to segment the dataset. When the window moves backward, a series of sample data covering each other will be formed. In the selection of the sliding window width, we tried three, six, and nine sample points to predict the next sample point, and used the root mean square error (RMSE) as the scoring criterion for the prediction error, which is defined in (21). The results are shown in Table 6. It was finally determined that the width of the input window with the smallest prediction error was six. Therefore, six sample points were used to predict the next sample point.

(22) $$RMSE=\sqrt{\frac{1}{n}\sum_{i=1}^{n}{\left({\hat{y}}_{i}-y_{i}\right)}^{2}}$$

where $y_{i}$ is the true value and ${\hat{y}}_{i}$ is the predicted value.

#### 4.3.2. Testing Results

**Figure 5:**
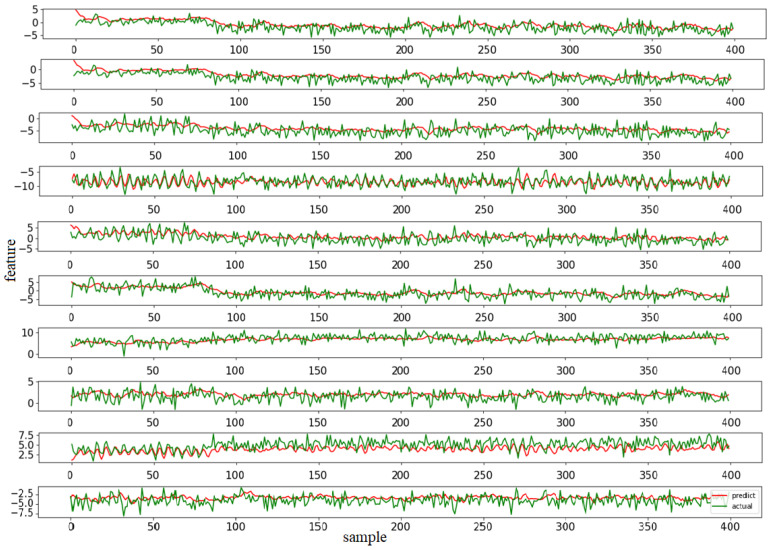
Comparison of prediction results (features 1 to 10 are shown in sequence).

During model training and evaluation stages, the RMSE was used as the loss function of the prediction model, and the prediction results are shown in Figure 5.

From Figure 5, it can be seen that the general feature trends can be predicted with certain errors. The reason for this is that the IMS dataset we used was designed for RUL prediction instead of degradation period prediction [25]. The final task of this paper was to classify the predicted features, which allows for an acceptable prediction error.

#### 4.3.3. Comparison with Other Models

In order to verify the prediction effect of the proposed attention-Bi-LSTM model, LSTM, Bi-LSTM, and attention-LSTM were applied to the IMS dataset for comparison. The comparative results are shown in Table 7. Since the attention mechanism can redistribute the proportions between sequences according to the predicted target, the use of a prediction model with an attention mechanism was able to improve the accuracy. Moreover, Bi-LSTM was able to capture features of both past and upcoming time series, and the performance of Bi-LSTM was also better than that of a single LSTM. We can see that the best prediction model was attention-Bi-LSTM (1.818/1.686).

### 4.4. Classification

**Figure 6:**
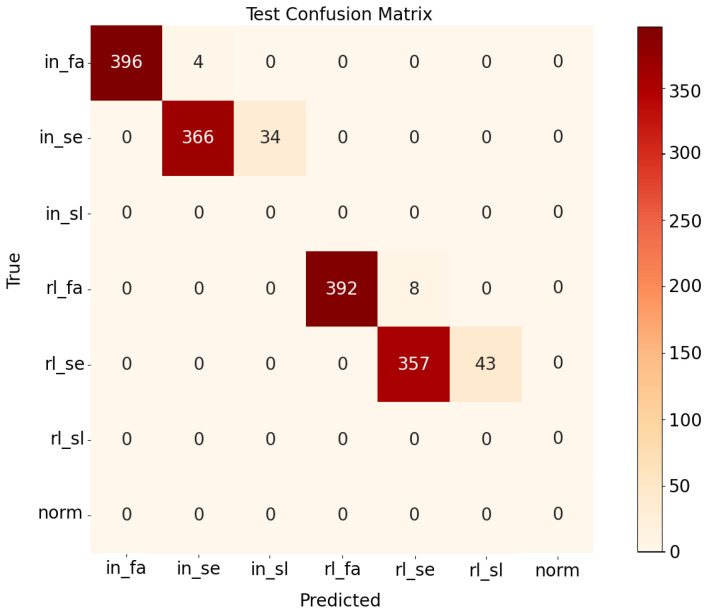
Confusion matrix of failure prediction results(“in” and “rl” denote inner and roller element defects, respectively; “sl”, “se” and “fa” represent slight, severe, and failure periods).

After obtaining the spatiotemporal features and the degradation trends of the bearing, the SVC was used to identify the degradation period and fault type. In this step, the modes of classification of the severe period and failure period for inner race and roller element faults are presented, which is more significant than normal mode identification. In this simulation, the data were collected every ten minutes, and our task was to realize the identification of the degradation period and fault type for the future 50 min. The classification results are shown in Figure 6.

As seen in the confusion matrix in Figure 6, the classification accuracy of the proposed SVC model combined with the attention-Bi-LSTM prediction model can reach 0.944. Furthermore, the classification accuracy of the failure mode can even reach 0.985. According to the background running data, it can be found that samples with classification errors are distributed at the connection of two adjacent periods, whereas the other samples are almost classified correctly. In summary, we can achieve short-term predictions of failure types and degradation periods through the use of our proposed fault prediction method.

**Remark** **1.** *The time-series signals in each window have one class label. The window size is determined using trial and error with a simulation method. The final accuracy of the prediction results with different window sizes is listed in Table 8. Table 8 shows a comparison of the test accuracy, sampling time, and test time of the proposed fault prediction method with three different window sizes. From Table 8, it can be seen that under the window size of 256, the test accuracy of the proposed method was higher than that of 2048 and 4096. This is because when the window size is larger, the number of windows is fewer. This directly leads to a lack of training set data in the feature prediction stage, especially for severe and failure periods, and the key information cannot be captured during feature prediction. As a result, the final classification accuracy of the predicted features would be lower. On the other hand, although the test time with a window size of 256 was about 0.01 s more than the other two cases, the fault prediction accuracy was about 0.1 higher. Therefore, the window size was chosen to be 256 in this simulation. When the programming environment and CPU change, the fault prediction time will also change accordingly.*

## 5. Conclusions

In this study, we divided the fault prediction task into three stages: feature extraction, feature prediction, and fault mode classification. In the first stage, the spatiotemporal features of the degradation period and fault mode are extracted through CNN-LSTM, based on vibration signals from multiple sensors. In the second stage, the features are sent to a Bi-LSTM network with an added attention mechanism to predict the feature trends. The Bi-LSTM method can take into account two-way sequences, and the attention mechanism can make the Bi-LSTM network work more efficiently by adjusting the weights. Finally, an SVC is used to classify the predicted spatiotemporal features of the deterioration period and fault type simultaneously. The IMS dataset was used to illustrate the effectiveness of the proposed fault prediction method. The simulation results show that a short-term prediction of deterioration period failure modes was achieved using the established fault prediction model. This would be helpful in arranging a maintenance plan in an industrial production setting.

The efficiency of the proposed three-stage fault prediction method is based on the premise that the training set and test set obey the same distribution, with plenty of samples. However, in real engineering scenarios, rolling bearings usually work in normal conditions under different work conditions, which leads to different distributions and few fault data. Therefore, fault prediction strategies should be investigated considering these problems in the future work.

## Figures and Tables

**Table 1:** Introduction to dataset 1.

Dataset	Bearing	Fault Type	Sensor Number
	1-1	-	2
dataset1	1-2	-	2
	1-3	inner race defect	2
	1-4	roller element defect	2

**Table 2:** Degradation period settings.

File Numbers	Period	Norm	Slight	Severe	Failure
Bearing					
1-3 H		1–1850	1851–2119	2120–2151	2152–2156
1-3 V		1–1850	1851–2119	2120–2151	2152–2156
1-4 H		1–1600	1601–2128	2129–2151	2152–2156
1-4 V		1–1600	1601–2128	2129–2151	2152–2156

**Table 3:** Network parameter settings in the feature extraction stage.

Layer	Type	Kernel Size/Stride/Numbers	Activation Function	Padding	BN
1-1	Sensor H	-	-	-	N
1-2	Sensor V	-	-	-	N
2-1	1D Convolution	64/16/16	RELU	same	Y
2-2	1D Convolution	64/16/16	RELU	same	Y
3-1	1D Maxpooling	2/2	-	valid	N
3-2	1D Maxpooling	2/2	-	valid	N
4-1	1D Convolution	32/8/32	RELU	same	Y
4-2	1D Convolution	32/8/32	RELU	same	Y
5-1	LSTM	100	Tanh/Sigmoid	-	N
5-2	LSTM	100	Tanh/Sigmoid	-	N
6-1	LSTM	40	Tanh/Sigmoid	-	N
6-2	LSTM	40	Tanh/Sigmoid	-	N
7	Concatenate	-	-	-	N
8	Dense1	128	-	-	N
9	Dense2	10(feature)	-	-	N
10	Softmax	-	-	-	N
11	Cross-entropy	-	-	-	N

**Table 4:** Comparison of feature extraction model with different sensor types.

Sensor Type	Accuracy
Sensor H	0.892
Sensor V	0.832
**Sensor H and Sensor V**	**0.928**

**Table 5:** Predictive model network parameter settings.

Layer	Units	Activation Function
Input	-	-
Bi-LSTM	100	Tanh/Sigmoid
Attention	-	-
Dense	75	RELU
Dense	1	-

**Table 6:** The impact of different input window widths on the prediction results.

Input	RMSE
Three inputs	2.221
**Six inputs**	**1.818**
Nine inputs	1.906

**Table 7:** Comparison of prediction models.

Algorithm	RMSE
LSTM	1.875
Bi-LSTM	1.828
Attention-LSTM	1.838
**Attention-Bi-LSTM**	**1.818**

**Table 8:** Window size and its influence on failure prediction.

Window Size × the Number of Windows	The Accuracy of Fault Mode Prediction	Sampling Time (s)	Fault Prediction Time (s)
4096 × 5	0.8	0.2	0.244
2048 × 10	0.86	0.1	0.248
**256 × 80**	**0.944**	**0.0125**	**0.255**

## Data Availability

Not applicable.
